# Application of OpenArray Technology to Assess Changes in the Expression of Functionally Significant Genes in the Substantia Nigra of Mice in a Model of Parkinson’s Disease

**DOI:** 10.3390/genes14122202

**Published:** 2023-12-12

**Authors:** Dmitry Troshev, Anna Kolacheva, Ekaterina Pavlova, Victor Blokhin, Michael Ugrumov

**Affiliations:** Laboratory of Neural and Neuroendocrine Regulations, Koltzov Institute of Developmental Biology of the Russian Academy of Sciences, 119334 Moscow, Russia; dmitry.vad.troshev@gmail.com (D.T.); annakolacheva@gmail.com (A.K.); guchia@gmail.com (E.P.); victor.blokhin@hotmail.com (V.B.)

**Keywords:** substantia nigra, mice, model of Parkinson’s disease, MPTP, reference genes, OpenArray

## Abstract

Studying the molecular mechanisms of the pathogenesis of Parkinson’s disease (PD) is critical to improve PD treatment. We used OpenArray technology to assess gene expression in the substantia nigra (SN) cells of mice in a 1-methyl-4-phenyl-1,2,3,6-tetrahydropyridine (MPTP) model of PD and in controls. Among the 11 housekeeping genes tested, *Rps27a* was taken as the reference gene due to its most stable expression in normal and experimental conditions. From 101 genes encoding functionally significant proteins of nigrostriatal dopaminergic neurons, 57 highly expressed genes were selected to assess their expressions in the PD model and in the controls. The expressions of *Th*, *Ddc*, *Maoa*, *Comt*, *Slc6a3*, *Slc18a2*, *Drd2*, and *Nr4a2* decreased in the experiment compared to the control, indicating decreases in the synthesis, degradation, and transport of dopamine and the impaired autoregulation of dopaminergic neurons. The expressions of *Tubb3*, *Map2*, *Syn1*, *Syt1*, *Rab7*, *Sod1*, *Cib1*, *Gpx1*, *Psmd4*, *Ubb*, *Usp47*, and *Ctsb* genes were also decreased in the MPTP-treated mice, indicating impairments of axonal and vesicular transport and abnormal functioning of the antioxidant and ubiquitin-proteasome systems in the SN. The detected decreases in the expressions of *Snca*, *Nsf*, *Dnm1l*, and *Keap1* may serve to reduce pathological protein aggregation, increase dopamine release in the striatum, prevent mitophagy, and restore the redox status of SN cells.

## 1. Introduction

Parkinson’s disease (PD) is a socially significant neurodegenerative disorder, ranking second in incidence and severity after Alzheimer’s disease. It is characterized by the progressive degeneration of dopaminergic (DAergic) neurons in the substantia nigra (SN), a key regulator of motor function [1,2]. The number of patients with PD is growing rapidly [3] and is expected to increase from 4.1 million in 2005 to almost 8.7 million by 2030 [4]. PD diagnosis relies on the manifestation of specific motor symptoms (rigidity, bradykinesia, and tremor) [5,6,7,8], when up to 60% of DAergic neurons die in the SN, and the level of dopamine (DA) in the striatum decreases by 70–80% [1,9,10,11]. After the diagnosis of PD, patients are treated symptomatically with drugs containing L-DOPA and dopamine agonists. However, this treatment has limited effectiveness, since it does not slow down the progression of the disease and does not prevent the disability of patients [5,7,12,13,14,15].

From the above, it follows that one of the objectives facing neurologists, neuropharmacologists, and neurophysiologists is to improve the current treatment of PD. Based on the translational medicine paradigm, this is possible by elucidating the molecular mechanisms of neurodegeneration and neuroplasticity. Along with genetic mouse models of PD, treatment with neurotoxins is widely used for these investigations [16,17,18,19]. The most commonly used models of this kind are based on the systemic administration of 1-methyl-4-phenyl-1,2,3,6-tetrahydropyridine (MPTP), the neurotoxin of DAergic neurons [17]. The molecular mechanisms of PD pathogenesis are studied in this model at three interconnected levels, evaluating the gene expressions of functionally significant proteins of nigrostriatal DAergic neurons [20,21,22] as well as the content and functional activities of these proteins [23,24,25].

The number of identified genes enco”Ing’proteins involved in the pathogenesis of PD is gradually increasing [25,26,27,28]. Moreover, in PD, the disruption of various metabolic systems in DAergic neurons of the SN, such as the protein degradation system and the antioxidant system, and the regulation of neurotrophic factors have been shown [29,30,31,32]. Therefore, when developing disease models and when testing potential drugs, it is necessary to evaluate the expressions of numerous genes related to different intracellular processes. In this context, the development of high-performance PCR methods for the simultaneous assessment of the expressions of many genes in individual small samples is of particular value [33,34,35].

OpenArray, developed by Thermo Fisher Scientific, is one of the high-performance RT-qPCR technologies [33]. A specific characteristic of this technology is that for each sample, gene amplification is carried out on a chip in a large number of microscopic arrays. Compared to RT-qPCR, microvolume amplification ensures high efficiency of the method with significant saving in reagents and time spent. Each chip can simultaneously assess the expressions of up to 112 genes in 24 samples. At the request of consumers, the manufacturer produces chips with a required set of primers, which, on the one hand, ensures the reproducibility of results obtained in different laboratories, and on the other hand, allows for the development of target chips for specific diagnostic or research purposes.

Even the detection of small changes in the gene expressions of functionally significant proteins is important for elucidating the molecular mechanisms of the pathogenesis of any disease [36]. To analyze the RT-qPCR results, it is necessary to know which genes can serve as reference genes (RGs). Indeed, the expressions of genes of interest should be assessed in relation to the reference ones. The expressions of reference genes should not reliably change in pathology, for example, during neurodegeneration in PD. They are called housekeeping genes (HKGs) [36,37], which are expressed in all cells of the body, and the proteins they encode are necessary for the normal functioning of the cell. However, the expressions of some HKGs may still change during neurodegeneration [38], which requires preliminary testing of the stability of HKG expression in each specific pathology. In addition, it should be taken into account that the most stable HKGs may differ in various brain regions in patients with PD and in mice models of PD [37]. In this regard, the choice of HKGs that will be used for comparison with the expressions of genes of interest is crucial for the correct interpretation of the RT-qPCR data [36].

Proceeding from the above, this study aimed to analyze the expressions of numerous genes encoding functionally significant proteins in SN cells in mice in normal conditions and in PD models using OpenArray. To carry this out, it was necessary to meet the following objectives: (1) select the most stably expressed HKGs based on the assay of their expressions in mice under normal conditions and in PD modeling and (2) evaluate changes in the expressions of numerous highly expressed genes in the SN of a mouse PD model.

## 2. Materials and Methods

### 2.1. Animals and Experimental Procedures

We used male C57BL/6 mice (*n* = 16) aged 8–12 weeks and weighing 22–25 g, obtained from the Stolbovaya nursery (SKMT RAMS, Stolbovaya, Moscow region, Russia). Animals were kept in standard laboratory vivarium conditions at a temperature of 21–23 °C and a light cycle of 12:12 h, and they had free access to food and water.

To model the clinical stage of PD (hereinafter referred to as PD modeling), mice were subcutaneously injected with MPTP (Sigma-Aldrich, St. Louis, MO, USA) 4 times at a single dose of 12 mg/kg with an interval of 2 h between injections (*n* = 8) [16]. The control group of animals was administered a 0.9% NaCl solution according to the same scheme (*n* = 8).

### 2.2. Collection of Biological Material

Biological material was collected two weeks after the administration of MPTP or 0.9% NaCl. Mice were anesthetized with isoflurane (Baxter, Deerfield, IL, USA) in a SomnoSuite anesthesia machine (Kent Scientific Corporation, Torrington, CT, USA), decapitated, and the brains were removed and cut along the midsagittal plane at a cold temperature. The striatum and SN were then excised from the brain under the control in a Leica M60 stereomicroscope (Leica Microsystems, Wetzlar, Germany). The locations of the striatum and SN were determined using the mouse brain atlas [39]. Striatum samples were weighed, and the striatum and SN were frozen in liquid nitrogen and stored at −70 °C until processing for high-performance liquid chromatography with electrochemical detection (striatum) or RNA extraction (SN).

### 2.3. High-Performance Liquid Chromatography with Electrochemical Detection

High-performance liquid chromatography with electrochemical detection was used to determine the concentration of DA and DA metabolites in the striatum samples as previously described [24].

### 2.4. Reverse Transcription Quantitative Real-Time Polymerase Chain Reaction

Extraction of total RNA from the SN samples of mice in all groups and cDNA synthesis were carried out as described in our previous work [22].

RT-qPCR analysis was carried out using TaqMan Open Array RT PCR Custom Format 112 chips (Lot 37B6745, REF 4470756 Applied Biosystems, Waltham, MA, USA). This study assessed the expressions of 101 genes of interest and 11 HKGs (Table S1). For RT-qPCR, 250 ng/µL cDNA was taken. Raw data analysis was performed using the QuantStudio 12K Flex software (version 1.3, Applied Biosystems, Waltham, MA, USA) and Excel (Microsoft Corporation, Redmond, WA, USA).

The stability of HKG expression in the SN under normal conditions and when modeling PD was assessed using the RefFinder program [40] based on the algorithms of the GeNorm [41], NormFinder [42], and BestKeeper programs [43] and the comparative ΔCt method [44]. This allowed us to rank stably expressed genes. Using the above algorithms, RefFinder was used to calculate the score for each gene and the geometric mean of their scores to determine the final gene ranking (https://blooge.cn/RefFinder/?type=reference, accessed on 23 October 2023). Stability of gene expression was assessed using a composite score for each gene in control and PD modeling. The closer the score is to 1, the more stable the gene expression is considered. For each HKGs, the total score was calculated as the sum of the comprehensive ranking values in the control and PD modeling.

Gene expression levels are expressed as 2^−ΔΔCt^ values normalized to the expression of *Rps27a* as an RG. Formulas (1) and (2) were used for calculating ΔΔCt as follows:

∆Ct = (Ct(*gene*) − Ct(*Rps27a*)) (1)

∆∆Ct = (∆Ct(sample) − ∆Ct(medium control)) (2)

The results were calculated as the geometric mean of the group [45].

### 2.5. Statistics

Statistical analysis was carried out using GraphPad Prism 9 software (version 9.5.1, GraphPad Software, Inc., La Jolla, CA, USA). The normality of the groups was assessed using the Shapiro–Wilk test. For pairwise comparison, the unpaired Student’s *t*-test was used. The results are presented as mean ± SEM. Differences were considered significant at *p* ≤ 0.05.

## 3. Results and Discussion

### 3.1. Characteristics of the Parkinson’s Disease Model

We used an acute mouse model of the clinical stage of PD that was previously developed in our laboratory [16]. This model reproduces the landmarks of the state of the nigrostriatal DAergic system in patients after the appearance of specific motor symptoms and diagnosis of the disease [1]. These include a decrease in the level of DA in the striatum by more than 70–80%, a loss of almost half of the DAergic neurons in the SN, and impaired motor behavior [16].

**Figure 1:**
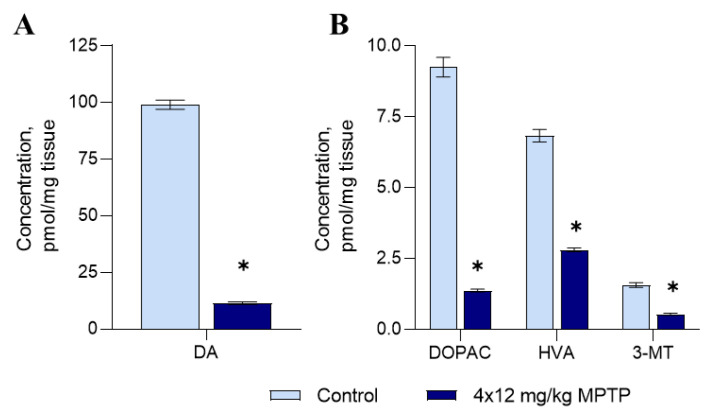
Concentrations of dopamine (DA) (**A**), 3,4-dihydroxyphenylacetic acid (DOPAC), homovanillic acid (HVA), and 3-methoxytyramine (3-MT) (**B**) in the striatum in a mouse model of Parkinson’s disease. The Shapiro–Wilk test was used to assess the normal distribution of the groups. Statistics indicate significance via the unpaired *t* -test (* *p* ≤ 0.05 compared with the control group). Data are presented as mean ± SEM; *n* = 8 for each group.

The correct reproduction of the PD model was tested in this work by the crucial indicator—a threshold decrease in the level of DA in the striatum. According to our data, the concentration of DA in the striatum of mice in the control group was 99 pmol/mg, and after the administration of MPTP, it decreased to 12 pmol/mg, which was 12% of the control level (Figure 1A). These data indicate the correct reproduction of the PD model.

Concentrations of DA metabolites, including 3,4-dihydroxyphenylacetic acid (DOPAC), 3-methoxytyramine (3-MT), and homovanillic acid (HVA), decreased in the striatum of MPTP-treated mice by 85%, 66%, and 59%, respectively (Figure 1B).

### 3.2. Selection of Stably Expressed Housekeeping Genes Based on Analysis of Their Expression in Normal Conditions and in Modeling Parkinson’s Disease

Based on previous experience using RG in RT-qPCR [36,37,46,47,48,49,50], we selected 11 HKGs: X-prolyl aminopeptidase (aminopeptidase P) 1 (*Xpnpep1*), alanyl-tRNA synthetase (*Aars*), GTPase activating protein and VPS9 domains 1 (*Gapvd1*), oxysterol binding protein (*Osbp*), glyceraldehyde-3-phosphate dehydrogenase (*Gapdh*), succinate dehydrogenase complex, subunit A (*Sdha*), 40S ribosomal protein S27a (*Rps27a*), ubiquitin-conjugating enzyme E2D2 (*Ube2d2*), cytochrome C1 (*Cyc1*), ribosomal protein L13 (*Rpl13*), and hypoxanthine guanine phosphoribosyl transferase (*Hprt*). The stability of the expressions of some genes was previously shown in patients with PD and in animal models of PD [36,37,49]. However, the expressions of these genes have not always been assessed in SN [36,46], including SN in the mouse model of PD used in this work.

The stability of HKG expression in the SN of mice of the control and experimental groups was assessed using four methods proposed in RefFinder (comparative method ΔCt, BestKeeper, NormFinder, and GeNorm) (Table 1).

We have shown that among the selected HKGs, *Rps27a* has the most stable expression in both normal conditions and in the PD model. In addition to this gene, we identified five additional genes whose expressions were quite stable during neurodegeneration: *Ube2d2a*, *Hprt*, *Cyc1*, *Sdha*, and *Rpl13*. It is noteworthy that two of the six genes we selected, *Cyc1* and *Rpl13*, were previously used as RGs to assess gene expression with RT-qPCR in autopsy brain material from patients suffering from neurodegenerative diseases [36].

Based on the results obtained, we chose *Rps27a* as an RG to evaluate gene expression in the SN of a mouse model of PD. It should be noted that when choosing an RG, it is necessary to take into account what tissue, human pathology, and pathology models will be tested. For example, according to our data obtained on SN in the mouse model of PD, and according to the data obtained in a study of human skin cancer [50], *Rps27a* is considered a stably expressed gene that can be used as an RG in RT-qPCR. On the contrary, in multiple sclerosis, the expression of *Rps27a* changes, and it is considered a biomarker of the disease [51].

### 3.3. Assessing the Gene Expression Using OpenArray and Developing a Panel of Highly Expressed Genes

When developing the initial panel of genes of interest, we relied on previously obtained information on the molecular mechanisms of neurodegeneration and neuroplasticity, including changes in the gene expressions of functionally significant proteins, in the SN of patients with PD [28,52,53,54,55,56,57,58,59]. As a result, the initial panel consisted of 101 genes of proteins involved in the functioning of SN neurons and the pathogenesis of PD or, in other words, in the mechanisms of neurodegeneration and neuroplasticity.

The genes were collected into clusters according to the functions of the proteins they encode:

1. DA synthesis and degradation: Tyrosine hydroxylase (*Th*), dopa decarboxylase (*Ddc*), dopamine β-hydroxylase (*Dbh*), phenylethanolamine N-methyltransferase (*Pnmt*), monoamine oxidase A and B (*Maoa* and *Maob*), and catechol-O-methyltransferase (*Comt*);
2. DA transport, DA receptors, and transcriptional factors: DA transporter (*Slc6a3*), vesicular monoamine transporter 1 and 2 (*Slc18a1* and *Slc18a2)*, plasma membrane monoamine transporter (*Slc29a4*), DA receptors 1–5 types (*Drd1–Drd5*), nuclear receptor subfamily 4 group A member 2 (Nurr1, *Nr4a2*); and paired-like homeodomain 3 (*Pitx3*);
3. Axonal transport and microtubules: Kinesin (*Kif1a*, *Kif1b*, *Kif5a*, and *Kif2c*), dynein (*Dync1h1* and *Dynll1*), dynactin 1 (*Dctn1*), τau-protein (*Mapt*), microtubule-associated protein 2 (*Map2*), MAP/microtubule affinity regulating kinase 2 (*Mark2*), and tubulin (*Tubb3*, *Tuba1a*);
4. Vesicle cycle for neurotransmission: α-synuclein (*Snca*), synapsin 1 (*Syn1*), syntaxin 1A (*Stx1a*), synaptotagmin 1 and 11 (*Syt1*, *Syt11*), Rab protein 5a и 7 (*Rab5a*, *Rab7*), N-ethylmaleimide sensitive fusion protein (*Nsf*), dynamin 1-like protein (*Dnm1l*), and vacuolar protein sorting ortholog 35 (*Vps35*);
5. Neuroprotection: Superoxide dismutase 1 (*Sod1*), glutathione peroxidase 1 (*Gpx1*), glutathione reductase (*Gsr*), thioredoxin reductase 1 (*Txnrd1*), nitric oxide synthase 1 (*Nos1*), peroxiredoxin 1 (*Prdx1*), nuclear factor erythroid 2-related factor 2 (*Nfe2l2*), angiotensin II receptor type 2 (*Agtr2*), sigma-1 receptor (*Sigmar1*), kelch-like ECH-associated protein 1 (*Keap1*), brain-derived neurotrophic factor (*Bdnf)*, glial cell-derived neurotrophic factor (*Gdnf*), nerve growth factor (*Ngf*), vascular endothelial growth factor A (*Vegfa*), cerebral DA neurotrophic factor (*Cdnf*), neurotrophic tyrosine kinase receptor types 1 and 2 (*Ntrk1* and *Ntrk2*), nerve growth factor receptor (*Ngfr*), matrix metalloproteinase-3 (*Mmp3*), Wnt family member 11 (*Wnt11*), catenin β-1 (*Ctnnb1*), and calbindin 1 (*Calb1*);
6. Protein degradation: Calcium channel voltage-dependent L type alpha 1D subunit (*Cacna1d*), transient receptor potential cation channel subfamily M member 2 (*Trpm2*), E3 ubiquitin ligase (Parkin) (*Park2*), ubiquitin-conjugating enzyme E2N (*Ube2n*), ubiquitin-like modifier activating enzyme 3 (*Uba3*), proteasome 20S subunit beta 4 (*Psmb4*), proteasome 26S subunit ATPase 3 (*Psmc3*), proteasome 26S subunit non-ATPase 4 (*Psmd4*), ubiquitin-specific peptidase 47 (*Usp47*), ubiquitin B (*Ubb*), and cathepsin B (*Ctsb*);
7. Cell death: Caspases 1 and 3 (*Casp1* and *Casp3*), poly [ADP-ribose] polymerase 1 (*Parp1*), apoptosis-inducing factor mitochondria associated 1 (*Aifm1*), calcium and integrin binding 1 (*Cib1*), transformation-related protein 53 (*Trp53*), Bax protein (*Bax*), c-Fos protein (*Fos*), mitogen-activated protein kinase 8 (*Mapk8*), lysosomal-associated membrane protein 2 (*Lamp2*), autophagy-related 16-like 1 and 5 (*Atg16l1* and *Atg5*), calpain-1 (*Capn1*), tumor necrosis factor (*Tnf*), endoplasmic reticulum to nucleus signaling 2 (*Ern2*), eukaryotic translation initiation factor 2-alpha kinase 3 (*Ef2ak3*), and activating transcription factor 6 (*Atf6*);
8. Inflammation and glial activation: Glial fibrillary acidic protein (*Gfap*), interferon gamma (*Ifng*), transforming growth factor beta 1 (*Tgfb1*), protein kinase B alpha (*Akt1*), cannabinoid receptor 1 (*Cnr1*), prostaglandin-endoperoxide synthase 2 (*Ptgs2*), CDC like kinase 1 (*Clk1*), transforming growth factor beta 1 (*Traf1*), C-X-C motif chemokine 11 (*Cxcl11*).

OpenArray, due to the use of a small sample volume in the microscopic arrays, is optimal for the evaluation of highly expressed genes when the amplification curve crosses the detection threshold before cycle 28 [60,61]. This is due to the fact that the probability of carrying out a PCR in each microscopic array in this case is at a maximum. It is methodologically incorrect to evaluate the expressions of lowly expressed genes (the amplification curve crosses the detection threshold between cycles 28 and 34) without additional preparatory procedures, as this leads to errors and a poor reproducibility of the results [33]. The preamplification of cDNA before OpenArray serves to reduce the cycle of crossing the detection threshold of the amplification curve (up to 10 cycles) without changing the results, which expands the range of analysis [34,62,63].

According to our data, in the SN, out of 101 genes included in the original panel, 57 have high levels of expression, 22 have low levels of expression, and we were unable to detect the expressions of 22 genes (Table 2). The latter may indicate the absence or very low expressions of these genes, in which the intersection of the amplification curve of the detection threshold exceeds 34 cycles. The lack of expression in the SN of genes such as *Dbh*, *Pnmt*, and *Slc18a1* was expected, since they encode enzymes for the synthesis of norepinephrine, adrenaline, as well as vesicular monoamine transporter 1, which is characteristic of neuroendocrine cells [64]. In the SN, we also failed to detect the expressions of the genes for three DA receptors (*Drd3*–*Drd5*), the expression of *Kif2c*, which is involved in the depolymerization of microtubules in dendrites and regulates the invasion of microtubules into neuron spines when the neuronal activity changes [65], and the expressions of a number of genes from the “neuroprotection”, “cell death”, and “inflammation and glial activation” clusters (Table 2).

Since OpenArray is best suited for assessing highly expressed genes, genes with low expressions were excluded from the final panel: *Slc29a4*, *Drd1*, *Kif1b*, *Mark2*, *Dynclh1*, *Stx1a*, *Vegfa*, *Bdnf*, *Nos1*, *Agtr2*, *Park2*, *Cacna1d*, *Trpm2*, *Casp1*, *Casp3*, *Map3k5*, *Fos*, *Capn1*, *Eif2ak3*, *Atf6*, *Atg16l1*, and *Tgfb1*. Thus, the final panel consisted of 57 genes with expressions that could be assessed in SN using OpenArray. However, we do not exclude that when modeling PD, the expression of some low-expressed genes encoding proteins involved in neuroprotection, cell death, inflammation, and glial activation may be higher than in animals in the control group.

### 3.4. Evaluation of Gene Expression in the Substantia Nigra in a Mouse Model of Parkinson’s Disease

The resulting panel, consisting of 57 genes, was used to study gene expression in SN in a mouse model of PD using OpenArray.

**Figure 2:**
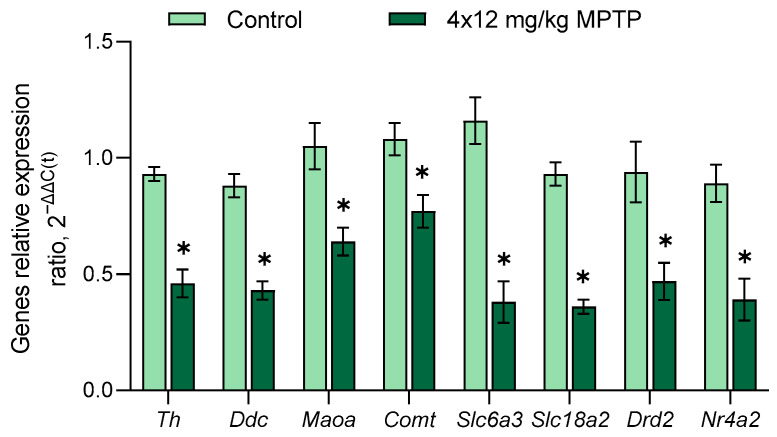
Changes in the expressions of genes encoding dopamine-synthesizing enzymes (*Th* and *Ddc*), dopamine-degrading enzymes (*Maoa* and *Comt*), dopamine transporters (*Slc6a3* and *Slc18a2*), dopamine receptor (*Drd2*), and transcription factor Nurr1 (*Nr4a2*) in the substantia nigra in a mouse model of Parkinson’s disease. The Shapiro–Wilk test was used to assess the normal distribution of the groups. Statistics indicate significance by the unpaired *t*-test (* *p* ≤ 0.05 compared with the control group). Data are presented as mean ± SEM; *n* = 8 for each group.

According to our data, in SN, the expressions of genes encoding DA-synthesizing enzymes (*Th* and *Ddc*) and DA transporters (*Slc6a3* and *Slc18a2*) are significantly reduced when modeling PD (Figure 2). At the same time, we showed that the expression of *Drd2*, encoding the D2 receptor, decreases in the SN in a model of PD. These data suggest a decreased synthesis of the D2 receptor, an autoreceptor of DAergic neurons. Previously, using the same PD model, we studied the expressions of the *Th*, *Ddc*, *Slc6a3*, *Slc18a2*, and *Drd2* genes in the SN [23,66,67] and in the sorted DAergic neurons of the SN [22]. The complete coincidence in the expressions of these genes means that both approaches (RT-qPCR and OpenArray technology) give the same results. The remaining genes in the developed panel were first assessed in the SN in a model of an early clinical stage of PD.

*Nr4a2* encodes the transcription factor Nurr1, which induces the expressions of genes of the DAergic phenotype in mesencephalic neurons [68,69]. *Th* expression is regulated directly by Nurr1, and the *Th* promoter has a binding site for Nurr1 [70,71]. This suggests a causal relationship between the decreased *Th* and *Nr4a2* expressions (Figure 2).

When modeling PD, we found a decrease in the expression of genes for enzymes that degrade DA: monoamine oxidase-A (*Maoa*) and catechol-O-methyltransferase (*Comt*) (Figure 2). DA is degraded in two stages and along two metabolic pathways; each one involves both enzymes [72,73]. In the first stage, monoamine oxidase-A and aldehyde dehydrogenase convert DA into DOPAC, and catechol-O-methyltransferase converts DA into 3-MT. In the second stage, catechol-O-methyltransferase converts DOPAC into HVA, and monoamine oxidase-A and aldehyde dehydrogenase convert DOPAC into 3-MT. This means that decreased expressions of *Maoa* and *Comt* may explain the decreased concentrations of DOPAC and 3-MT in the striatum. Considering that the concentration of DOPAC decreased to a greater extent than the concentration of 3-MT, we can conclude that DA degrades during PD modeling mainly in the extraneuronal space, where catechol-O-methyltransferase is localized [73]. In contrast to the monoamine oxidase-A gene, the expression of the monoamine oxidase-B gene (*Maob*) does not change in the PD model compared to the controls. This is probably explained by the fact that *Maoa* is mainly expressed in neurons, whereas *Maob* is expressed in astrocytes [74].

**Figure 3:**
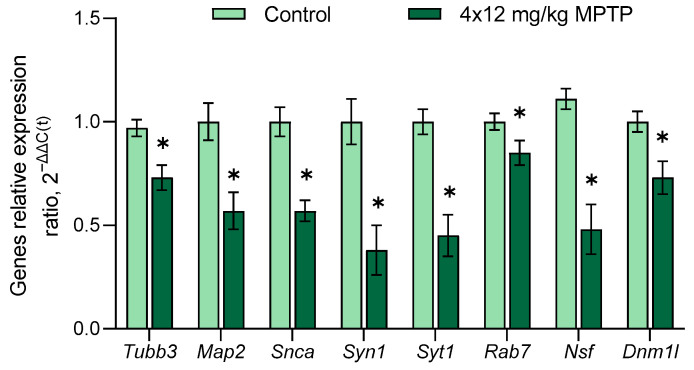
Changes in the expressions of genes for proteins associated with axonal transport (*Tubb3* and *Map2*) and the vesicular cycle (*Snca*, *Syn1*, *Syt1*, *Rab7*, *Nsf*, and *Dnm1l*) in the substantia nigra in a mouse model of Parkinson’s disease. The Shapiro–Wilk test was used to assess the normal distribution of the groups. Statistics indicate significance by the unpaired *t*-test (* *p* ≤ 0.05 compared with the control group). Data are presented as mean ± SEM. *n* = 8 for each group.

When assessing the expressions of genes encoding axonal transport proteins, we showed a decrease in the expressions of *Map2* and *Tubb3* in the SN of mice in a model of PD (Figure 3). Microtubule-associated protein-2, encoded by the *Map2* gene, is localized primarily in neuronal dendrites. This protein stabilizes the assembly of microtubules and ensures their interaction with other components of the neuronal cytoskeleton [75,76,77]. In addition, in PD patients, this protein induces the formation of fibrous aggregates and crystal-like structures within the nuclei of neurons. It also colocalizes with α-synuclein and ubiquitin in cytoplasmic inclusion bodies [75]. β3-tubulin is one of the structural proteins of microtubules, and mutations in the *Tubb3* can lead to the impaired production of α/β heterodimers and thereby lead to a decrease in the stability of microtubules and the disruption of axonal transport [78]. β3-tubulin found in the bloodstream is considered one of the nonspecific biomarkers of neurodegenerative diseases, including Alzheimer’s disease and PD [79]. Decreases in the expressions of genes for proteins, including tubulins, kinesins, and dyneins, which are involved in anterograde and retrograde axonal transport, were previously shown in DAergic neurons of the SN of patients with PD [52]. Impaired axonal transport is considered an important characteristic of this disease [80,81]. Our results show that axonal transport is also impaired in PD models, as manifested by decreased expressions of genes encoding microtubule structural proteins and proteins regulating microtubule stability in the SN. This can lead to the disruption of the transport of organelles (mitochondria) and individual functionally important molecules along axons, promoting axonal degradation and neuronal death.

When assessing the expressions of genes encoding vesicular cycle proteins in the SN in a mouse model of PD, we observed decreases in the expressions of the following genes: *Snca*, *Syn1*, *Syt1*, *Rab7*, *Nsf*, and *Dnm1* (Figure 3). The *Snca* gene encodes α-synuclein, the presynaptic protein [82], and mutations of this gene lead to the development of PD [83,84]. The pathogenic neurotoxin is represented by prefibrillar α-synuclein, but not deposits of this protein—Lewy bodies. Oligomers or protofibrils of α-synuclein disrupt the normal degradation of proteins in the cell, which negatively affects the functioning of organelles such as the mitochondria and the endoplasmic reticulum [85,86]. It should be noted that α-synuclein oligomers can spread from neuron to neuron in a prion-like fashion via the intercellular space, thereby expanding the zone of neurodegeneration [82,85]. The decrease in *Snca* expression in the SN that we discovered, which was also shown in other studies when modeling PD in mice [87], may lead to a decrease in protein aggregation.

When studying the molecular mechanisms of the PD pathogenesis, the evaluation of DA neurotransmission, which is largely provided by vesicular cycle proteins, is of great importance. The *Syn1* gene encodes synapsin 1, a protein involved in the transport of synaptic vesicles and contributes to the regulation of synaptogenesis and axonogenesis [88]. The decrease in the expression of this gene in the SN in a mouse model of PD that we discovered may indicate a disruption in the transport of synaptic vesicles and, thus, synaptic neurotransmission. This was previously shown in a subchronic mouse model of the clinical stage of PD [89]. Another vesicular cycle protein is synaptotagmin 1, encoded by the *Syt1* gene. This protein is a calcium sensor involved in triggering the release of DA and other neurotransmitters from the synaptic terminals [90]. The decrease in *Syt1* expression that we found in the mouse PD model may suggest a decrease in DA neurotransmission in SN. The third vesicular cycle protein, encoded by the *Rab7* gene, regulates the transport of late endosomes and autophagosomes, and its overexpression prevents the accumulation of the mutant A53T α-synuclein that was shown in the cell culture and in PD models in *Drosophila melanogaster* and rats [91,92]. The decrease in *Rab7* expression that we discovered during the modeling of PD indicates a decrease in the ability of SN cells to degrade pathological proteins in autophagosomes.

In response to the DAergic denervation of the striatum, compensatory processes are activated, aimed at minimizing DA deficiency in this part of the brain by increasing the functional activity of surviving DAergic neurons of the SN [93] and increasing the release of DA from their axonal terminals located in the striatum [94]. It should be noted that the presynaptic N-ethylmaleimide-sensitive hybrid protein encoded by the *Nsf* gene plays a fundamental role in synaptic neurotransmission. This protein is an ATPase, which couples ATP hydrolysis to the disassembly of SNARE proteins, allowing them to be included in the next round of synaptic vesicle exocytosis [95]. Increased ATPase activity of the N-ethylmaleimide-sensitive fusion protein is observed when it is phosphorylated by leucine-rich repeat kinase 2 at threonine 645 in the ATP-binding pocket of the D2 domain [95]. An N-ethylmaleimide-sensitive fusion protein is involved in the pathogenesis of some inherited forms of PD. Indeed, the *LRRK2* G2019S mutation in PD results in increased leucine-rich repeat kinase 2 activity, which increases the frequency of phosphorylation of the N-ethylmaleimide-sensitive fusion protein and leads to its accumulation in toxic inclusion bodies [96]. Since DA neurotransmission in the striatum is characterized by both the complete and partial fusion of synaptic vesicles with the plasma membrane according to the “kiss-and-run” mechanism, promoting the release of a small amount of DA into the synaptic cleft [97,98], it can be assumed that the decrease in *Nsf* expression that we discovered in this work contributes to a decrease in the synthesis of N-ethylmaleimide-sensitive fusion protein. This, in turn, can lead to the slower disassembly of SNARE proteins and hence an increase in the amount of DA released into the synaptic cleft. We believe that a decrease in *Nsf* gene expression may be one of the compensatory processes that develop in the SN when modeling PD.

The genes we studied also include *Dnm1l*, encoded dynamin-related protein 1, which is involved in mitochondrial fission and mitophagy [99]. The loss of the ability of mitochondria to divide due to the removal of dynamin-related protein leads to the degradation of DAergic axonal terminals in the striatum and contributes to the preferential death of nigral DAergic neurons [100]. A number of researchers emphasize the importance of Drp-1-dependent mitochondrial fragmentation to protect cells from death caused by aggregated α-synuclein [101]. At the same time, it was shown that the inhibition of the synthesis of dynamin-related protein 1 in MPTP-treated mice leads to a weakening of the toxic effect of MPTP and a restoration of the normal level of DA release in the striatum [102]. Based on these data, the decrease in *Dnm1l* expression in the SN that we observed in this study in a mouse model of PD seems to be a compensatory process that prevents mitophagy and protects existing mitochondria from degradation.

**Figure 4:**
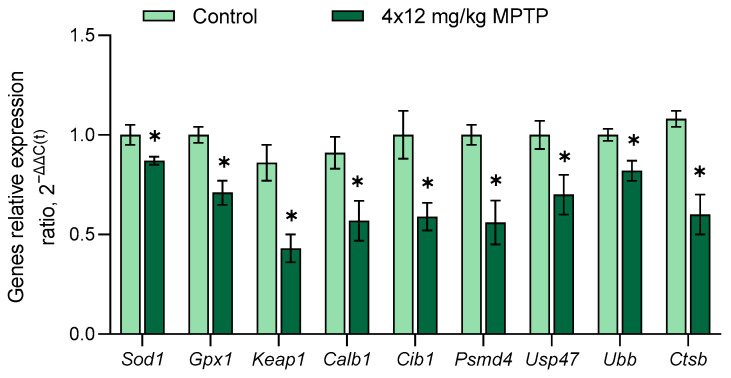
Changes in the expressions of genes encoding proteins of the antioxidant system (*Sod1* and *Gpx1*), transcription factors (*Keap1*), calcium-binding proteins (*Calb1* and *Cib1*) and proteins of the ubiquitin-proteasome system (*Psmd4*, *Ubb*, *Usp47*, and *Ctsb*) in the substantia nigra in a mouse model of Parkinson’s disease. The Shapiro–Wilk test was used to assess the normal distribution of the groups. Statistics indicate significance using unpaired *t* test (* *p* ≤ 0.05 vs. control group). Data are presented as mean ± SEM; *n* = 8 for each group.

It is well known that the death of neurons is caused by oxidative stress due to the increased production of highly reactive oxygen, highly reactive nitrogen, cations of certain metals, and a decreased activity of the antioxidant system, i.e., an impairment of the “redox status” of the cell [103]. When modeling PD, we found decreases in the expressions of the *Sod1* and *Gpx1* genes, encoding enzymes of the antioxidant system—superoxide dismutase type 1 and glutatione peroxydase 1 (Figure 4). This fact is consistent with the data on an increase in the level of oxidative stress that were obtained when studying an autopsy of the SN in PD patients [104,105]. In PD, a decrease in the glutathione content by 40% in the SN, but not in other parts of the brain, has also been shown [106,107,108]. Taken together, the above data indicate a selective decrease in the activity of the antioxidant system in DAergic neurons of the SN in PD [108].

We showed a decrease in the expression of the *Keap1* gene, encoding ECH Kelch-associated protein 1, associated with the transcription factor Nrf2b, in the SN of mice models of PD (Figure 4) [109,110]. Under oxidative stress or low *Keap1* expression, Nrf2 dissociates from ECH Kelch binding protein 1 and translocates to the nucleus, where it activates the expression of antioxidant system genes [111]. We assume that the decrease in *Keap1* expression is a compensatory process aimed at restoring the “redox status” of degenerating neurons.

When studying the mechanisms that contribute to the partial compensation of DA deficiency in the striatum when modeling PD, it is of particular interest to study the expressions of genes encoding calcium-binding proteins. Indeed, we found a decrease in the expression of *Calb1*, which encodes calbindin 1 protein. This protein binds calcium in the cytoplasm, protecting the cell from the cytotoxic effect of calcium [112]. It is logical to assume that the decrease in *Calb1* gene expression that we discovered, probably accompanied by a decrease in the synthesis of calbindin 1, may be a compensatory process aimed at stimulating DA release from striatal DAergic axons when modeling PD. However, it was previously shown that calbindin 1 regulates DA release and uptake in the ventral rather than the dorsal striatum, which is involved in the regulation of motor behavior [113]. Therefore, in the future, it will be desirable to elucidate the role of calbindin 1 in DA neurotransmission and compensatory processes in the dorsal striatum during the development of PD.

In addition, we showed a decrease in the expression of *Cib1* (Figure 4), encoding another calcium-binding protein, calcium- and integrin-binding protein 1, which has been shown to be involved in a wide range of intracellular processes, such as the regulation of microtubule formation during cell division [114] and the regulation of β-amyloid production by controlling the subcellular localization of γ-secretase [115]. It was previously shown that calcium- and integrin-binding protein 1 inhibits the activity of signal-regulating kinase 1, which prevents the apoptosis of DAergic neurons in the SN in PD modeling using 6-hydroxydopamine and MPTP [116,117]. In this regard, the decrease in the expression of the *Cib1* gene, which we found in the SN when modeling PD, may indicate a decrease in the ability of cells in this area of the brain to prevent apoptotic cell death.

It is well known that neurotrophic factors play important roles in the regulation of brain development and reparative processes in brain damage. This prompted the present study to evaluate the gene expressions of neurotrophic factors, neurotrophic factor receptors, and transcription factors involved in the activation of signaling pathways that may promote the survival of DAergic neurons in a mouse model of PD. However, according to our data, the expression of the *Ntrk2* gene, encoding the receptor for BDNF, does not change in the MPTP-treated mice. These data are in good agreement with previous pathological studies showing that the expression of *Ntrk2* does not change in patients with PD [53]. This is also the case for genes of neurotrophic factors, their receptors, and transcription factors involved in activation signaling pathways that promote the survival of DAergic neurons [52]. Thus, in PD patients and in animal models of clinical PD, there are no changes in the expressions of neurotrophic factors, neurotrophic factor receptors, and transcription factors involved in the activation of signaling pathways that may promote the survival of DAergic neurons. This indicates that with the significant death of SN DAergic neurons, which is characteristic of the clinical stage of PD, neurotrophic factors are not involved in the regulation of reparative processes associated with the degradation of the nigrostriatal DAergic system.

When evaluating the expressions of genes for proteins undergoing proteasomal and lysosomal degradation, we found decreased expressions of *Psmd4*, *Ubb*, *Usp47*, and *Ctsb* in the MPTP-treated mice (Figure 4). Since the ubiquitin–proteasome system ensures the degradation of misfolded and damaged proteins, its disruption leads to the accumulation of toxic proteins, including aggregated α-synuclein in PD [118]. The decreases in the expressions of genes encoding proteins of the 26S subunit of the proteasome (not ATPase 4) (*Psmd4*), ubiquitin-specific peptidase 47 (*Usp47*), ubiquitin B (*Ubb*), and cathepsin B (*Ctsb*) that we discovered in mice models of PD may be accompanied by decreased synthesis and impaired degradation of these proteins in SN cells. Our data are in good agreement with those showing decreases in the expressions of genes for proteins of the ubiquitin-proteasome system in nigral DAergic neurons in PD patients [52].

The mechanism of neuronal death depends on the regime of MPTP administration to mice, and the observed changes in gene expression depend on how long after exposure to the neurotoxin the analysis is carried out [67,119]. In acute models of PD, when MPTP is administered for one day, necroptosis predominates, while with subchronic or chronic regimes of neurotoxin administration, other types of cell death, mainly apoptosis, predominate [17,120,121,122]. In this case, DAergic neurons degenerate no more than two days after the administration of the neurotoxin [123,124]. Therefore, with the experimental design used (sample collection 2 weeks after MPTP administration), we did not expect to observe any changes in the expressions of genes associated with neuronal death.

## 4. Conclusions

Thus, in this study, among 11 HKGs, we selected *Rps27a* as the most stably expressed gene in the control and MPTP-treated mice. Of the 101 protein genes involved in the functioning of SN neurons in normal conditions and in PD modeling, 57 genes with high expressions were selected for their subsequent analyses using OpenArray technology. We showed decreases in the expressions of genes encoding proteins involved in the synthesis, degradation, transport of DA, and autoregulation of DAergic neurons. When modeling PD, the expressions of genes for proteins of axonal and vesicular transport, as well as proteins of the antioxidant and ubiquitin–proteasome systems, were also reduced in the SN. Simultaneous decreases in the expressions of *Snca*, *Nsf*, *Dnm1l*, and *Keap1* were shown in the MPTP-treated mice, which suggests the activation of compensatory processes in PD. These processes can serve to reduce the aggregation of pathological proteins, increase the release of dopamine in the striatum, inhibit mitophagy, and restore the “redox status” of SN cells. Undoubtedly, the knowledge about the molecular mechanisms of neurodegeneration and neuroplasticity of the nigrostriatal system that we obtained in a model of the early clinical stage of PD will be used for further research on this topic.

## Figures and Tables

**Table 1:** Ranking of the stability of the expressions of housekeeping genes in the substantia nigra in mice under normal conditions and in model of Parkinson’s disease, calculated using the RefFinder software (https://blooge.cn/RefFinder/?type=reference, accessed on 23 October 2023).

Gene Rank	Control					4 × 12 mg/kg MPTP					Summary Score
	Comprehensive Ranking	Delta CT	BestKeeper	NormFinder	GeNorm	Comprehensive Ranking	Delta CT	BestKeeper	NormFinder	GeNorm	
	HKG/Score	HKG/Score	HKG/Score	HKG/Score	HKG/Score	HKG/Score	HKG/Score	HKG/Score	HKG/Score	HKG/Score	HKG/Score
1	*Rps27a* 1.41	*Sdha* 0.57	*Rps27a* 0.333	*Sdha* 0.253	*Rps27a* 0.389	*Rps27a* 1.68	*Cyc1* 2.95	*Rpl13* 0.333	*Ube2d2a* 0.226	*Rps27a* 0.452	*Rps27a* 3.09
2	*Sdha* 2.45	*Rps27a* 0.57	*Gapdh* 0.417	*Rps27a* 0.267	*Ube2d2a* 0.389	*Ube2d2a* 1.97	*Rps27a* 3.01	*Rps27a* 0.417	*Rps27a* 0.226	*Ube2d2a* 0.452	*Ube2d2a* 5.95
3	*Hprt* 3.72	*Gapvd1* 0.59	*Aars* 0.456	*Gapvd1* 0.328	*Hprt* 0.402	*Rpl13* 2.28	*Rpl13* 3.05	*Ube2d2a* 0.583	*Rpl13* 0.311	*Rpl13* 0.583	*Hprt* 8.15
4	Gapvd1 3.87	*Hprt* 0.62	*Hprt* 0.5	*Hprt* 0.390	*Sdha* 0.437	*Cyc1* 3.16	*Hprt* 3.07	*Hprt* 0.889	*Cyc1* 1.197	*Hprt* 0.836	*Cyc1* 8.85
5	*Ube2d2a* 3.98	*Cyc1* 0.64	*Gapvd1* 0.583	*Cyc1* 0.415	*Gapvd1* 0.471	*Hprt* 4.43	*Ube2d2a* 3.1	*Cyc1* 1.056	*Xpnpep1* 1.655	*Cyc1* 0.979	*Sdha* 8.93
6	*Cyc1* 5.69	*Ube2d2a* 0.65	*Cyc1* 0.583	*Ube2d2a* 0.431	*Rpl13* 0.504	*Xpnpep1* 6.19	*Xpnpep1* 3.14	*Sdha* 1.306	*Hprt* 1.729	*Sdha* 1.084	*Rpl13* 9.24
7	*Aars* 6.26	*Rpl13* 0.67	*Ube2d2a* 0.583	*Rpl13* 0.476	*Cyc1* 0.530	*Sdha* 6.48	*Sdha* 3.21	*Xpnpep1* 1.403	*Sdha* 2.189	*Xpnpep1* 1.163	*Gapvd1* 13.87
8	*Gapdh* 6.69	*Aars* 0.71	*Rpl13* 0.611	*Aars* 0.538	*Aars* 0.560	*Gapdh* 8	*Gapdh* 3.33	*Gapdh* 1.667	*Gapdh* 2.216	*Gapdh* 1.259	*Gapdh* 14.69
9	*Rpl13* 6.96	*Xpnpep1* 0.73	*Sdha* *0.625*	*Xpnpep1* 0.571	*Xpnpep1* 0.580	*Aars* 9	*Aars* 3.42	*Aars* 1.75	*Aars* 2.593	*Aars* 1.303	*Aars* 15.26
10	*Xpnpep1* 9.24	*Gapdh* 0.78	*Xpnpep1* 0.625	*Gapdh* 0.623	*Gapdh* 0.616	*Gapvd1* 10	*Gapvd1* 8.95	*Gapvd1* 4.208	*Gapvd1* 8.064	*Gapvd1* 2.834	*Xpnpep1* 15.43
11	*Osbp* 11	*Osbp* 0.98	*Osbp* 0.75	*Osbp* 0.889	*Osbp* 0.682	*Osbp* 11	*Osbp* 11.72	*Osbp* 7.806	*Osbp* 11.389	*Osbp* 4.449	*Osbp* 22

HKG—housekeeping gene; GRV—geometric mean of ranking values. Gene Rank—ranking from most stable to least stable gene. Comprehensive ranking—geometric mean calculated using a score of four methods: comparative ∆CT, NormFinder, BestKeeper, and GeNorm. Summary score—sum of values (score) of comprehensive ranking for HKGs in mice in control and in the model of Parkinson’s disease.

**Table 2:** Protein genes whose expressions were assessed in the substantia nigra of mice using TaqMan OpenArray RT-PCR custom chips.

Gene Clusters	Highly Expressed Genes (Can Be Detected with OpenArray)	Genes with Low Expressions (Can Be Detected with OpenArray Following Pre-amplification)	Genes with Very Low Expressions or Genes Not Expressed (Cannot Be Detected with OpenArray)
Synthesis and degradation of dopamine	*Th*, *Ddc*, *Comt*, *Maoa*, *Maob*	*-*	*Dbh*, *Pnmt*
Dopamine transport, dopamine receptors, and transcription factors of dopaminergic neurons	*Slc18a2*, *Slc6a3*, *Drd2*, *Nr4a2*	*Slc29a4*, *Drd1*	*Drd3–Drd5*, *Slc18a1*, *Pitx3*
Axonal transport	*Tubb3*, *Tuba1a*, *Dynll1*, *Kif1a*, *Kif5a*, *Dctn1*, *Map2*, *Mapt*	*Kif1b*, *Dync1h1*, *Mark2*	*Kif2c*
Vesicular cycle and mediator release	*Syn1*, *Syt1*, *Snca*, *Syt11*, *Rab5a*, *Rab7*, *Dnm1l*, *Vps35*, *Nsf*,	*Stx1a*	*-*
Neuroprotection	*Gpx1*, *Gsr*, *Sod1*, *Prdx1*, *Txnrd1*, *Nfe2l2*, *Keap1*, *Sigmar1*, *Calb1*, *Ctnnb1*, *Ntrk2*	*Bdnf*, *Vegfa*, *Nos1*, *Agtr2*	*Ngf*, *Gdnf*, *Cdnf*, *Ntrk1*, *Mmp3*, *Wnt11*, *Ngfr*
Protein degradation	*Ubb*, *Uba3*, *Ube2n*, *Psmb4*, *Psmd4*, *Psmc3*, *Usp47*, *Ctsb*	*Park2*, *Cacna1d*, *Trpm2*	*-*
Cell death	*Parp1*, *Cib1*, *Aifm1Bax*, *Trp53*, *Lamp2*, *Mapk8*, *Atg5*	*Casp1*, *Casp3*, *Map3k5*, *Fos*, *Capn1*, *Eif2ak3*, *Atf6*, *Atg16l1*	*Tnf*, *Bcl2l11*, *Ern2*
Inflammation and glia activation	*Gfap*, *Clk1*, *Akt1*, *Cnr1*	*Tgfb1*	*Ifng*, *Cxcl11*, *Ptgs2*, *Traf1*

Gene names are indicated according to the National Library of Medicine GenBank (https://www.ncbi.nlm.nih.gov/genbank accessed on 4 November 2022).

## Data Availability

The data presented in this study are available upon request from the corresponding author. The data are not publicly available due to legal issues.

## Supplementary Materials

The following supporting information can be downloaded at https://www.mdpi.com/article/10.3390/genes14122202/s1, Table S1: Genes and their target names on RT-qPCR chips for OpenArray technology.

## Abbreviations

3-MT	3-methoxytyramine
DA	dopamine
DAergic	dopaminergic
DOPAC	3,4-dihydroxyphenylacetic acid
HKG	housekeeping gene
HVA	homovanillic acid
MPTP	1-methyl-4-phenyl-1,2,3,6-tetrahydropyridine
PD	Parkinson’s disease
RG	reference gene
RT-qPCR	Reverse-transcription quantitative real-time polymerase chain reaction
SN	substantia nigra
