# Supplementary material for: Application of OpenArray Technology to Assess Changes in the Expression of Functionally Significant Genes in the Substantia Nigra of Mice in a Model of Parkinson’s Disease
Source: Genes (Basel). 2023 Dec 12;14(12):2202. doi: 10.3390/genes14122202 (PMC10742853; doi:10.3390/genes14122202)
Supplement: Supplementary file 1 [file genes-14-02202-s001.zip › genes-2732998-supplementary.pdf]

Table S1. Genes and their target names on PCR chips for Open Array technology

No	Gene	Target name	Reporter
1	*Xpnpep1*	Mm00460040_m1	FAM
2	*Aars*	Mm00507627_m1	FAM
3	*Gapvd1*	Mm01336858_m1	FAM
4	*Osbp*	Mm01205197_m1	FAM
5	*Gapdh*	Mm99999915_g1	FAM
6	*Sdha*	Mm01352366_m1	FAM
7	*Rps27a*	Mm01180369_g1	FAM
8	*Ube2d2a*	Mm00785931_s1	FAM
9	*Cyc1*	Mm00470540_m1	FAM
10	*Rpl13*	Mm02526700_g1	FAM
11	*Gfap*	Mm01253033_m1	FAM
12	*Hprt*	Mm03024075_m1	FAM
13	*Th*	Mm00447557_m1	FAM
14	*Ddc*	Mm00516688_m1	FAM
15	*Dbh*	Mm00460472_m1	FAM
16	*Pnmt*	Mm00476993_m1	FAM
17	*Maoa*	Mm00558004_m1	FAM
18	*Maob*	Mm00555412_m1	FAM
19	*Comt*	Mm00514377_m1	FAM
20	*Slc6a3*	Mm00438388_m1	FAM
21	*Slc18a1*	Mm00461868_m1	FAM
22	*Slc18a2*	Mm00553058_m1	FAM
23	*Drd1*	Mm02620146_s1	FAM
24	*Drd2*	Mm00438545_m1	FAM
25	*Drd3*	Mm00432887_m1	FAM
26	*Drd4*	Mm00432893_m1	FAM
27	*Drd5*	Mm04210376_s1	FAM
28	*Kif1a*	Mm00492863_m1	FAM
29	*Kif1b*	Mm00801813_m1	FAM
30	*Kif5a*	Mm00515265_m1	FAM
31	*Kif2c*	Mm00728630_s1	FAM
32	*Dync1h1*	Mm00466548_m1	FAM
33	*Dynll1*	Mm00850282_g1	FAM
34	*Dctn1*	Mm01184845_m1	FAM
35	*Mapt*	Mm00521988_m1	FAM
36	*Map2*	Mm00485231_m1	FAM
37	*Mark2*	Mm01220150_g1	FAM
38	*Tubb3*	Mm00727586_s1	FAM
39	*Tuba1a*	Mm00846967_g1	FAM
40	*Snca*	Mm01188700_m1	FAM
41	*Syn1*	Mm00449772_m1	FAM
42	*Stx1a*	Mm00444008_m1	FAM
43	*Syt1*	Mm00436858_m1	FAM

44	*Syt11*	Mm00444517_m1	FAM
45	*Rab5a*	Mm00727887_s1	FAM
46	*Rab7*	Mm00784318_sH	FAM
47	*Nsf*	Mm00435390_m1	FAM
48	*Dnm1l*	Mm01342903_m1	FAM
49	*Vps35*	Mm00458167_m1	FAM
50	*Sod1*	Mm01344233_g1	FAM
51	*Gpx1*	Mm00656767_g1	FAM
52	*Gsr*	Mm00439154_m1	FAM
53	*Txnrd1*	Mm00443675_m1	FAM
54	*Nos1*	Mm01208059_m1	FAM
55	*Prdx1*	Mm01621996_s1	FAM
56	*Nfe2l2*	Mm00477784_m1	FAM
57	*Agtr2*	Mm00431727_g1	FAM
58	*Keap1*	Mm00497268_m1	FAM
59	*Sigmar1*	Mm01223547_g1	FAM
60	*Cacna1d*	Mm01209927_g1	FAM
61	*Trpm2*	Mm00663098_m1	FAM
62	*Park2*	Mm01323528_m1	FAM
63	*Ube2n*	Mm00779119_s1	FAM
64	*Uba3*	Mm00495866_m1	FAM
65	*Psmb4*	Mm01263563_m1	FAM
66	*Psmc3*	Mm00477177_m1	FAM
67	*Psmd4*	Mm01263490_m1	FAM
68	*Usp47*	Mm00659716_m1	FAM
69	*Ubb*	Mm01622233_g1	FAM
70	*Bdnf*	Mm04230607_s1	FAM
71	*Gdnf*	Mm00599849_m1	FAM
72	*Ngf*	Mm00443039_m1	FAM
73	*Vegfa*	Mm00437306_m1	FAM
74	*Cdnf*	Mm00617407_m1	FAM
75	*Ntrk2*	Mm00435422_m1	FAM
76	*Ntrk1*	Mm01219406_m1	FAM
77	*Ngfr*	Mm00446296_m1	FAM
78	*Nr4a2*	Mm00443060_m1	FAM
79	*Mmp3*	Mm00440295_m1	FAM
80	*Pitx3*	Mm01194166_g1	FAM
81	*Wnt11*	Mm00437327_g1	FAM
82	*Ctnnb1*	Mm00483039_m1	FAM
83	*Slc29a4*	Mm00525575_m1	FAM
84	*Calb1*	Mm00486647_m1	FAM
85	*Ifng*	Mm01168134_m1	FAM
86	*Tgfb1*	Mm01178820_m1	FAM
87	*Akt1*	Mm01331626_m1	FAM
88	*Cnr1*	Mm01212171_s1	FAM
89	*Ptgs2*	Mm00478374_m1	FAM
90	*Clk1*	Mm00438254_m1	FAM

91	*Traf1*	Mm00493827_m1	FAM
92	*Cxcl11*	Mm00444662_m1	FAM
93	*Casp1*	Mm00438023_m1	FAM
94	*Casp3*	Mm01195085_m1	FAM
95	*Parp1*	Mm01321084_m1	FAM
96	*Aifm1*	Mm00442548_m1	FAM
97	*Bcl2l11*	Mm00437796_m1	FAM
98	*Map3k5*	Mm00434883_m1	FAM
99	*Cib1*	Mm00501944_m1	FAM
100	*Trp53*	Mm01731290_g1	FAM
101	*Bax*	Mm00432051_m1	FAM
102	*Fos*	Mm00487425_m1	FAM
103	*Mapk8*	Mm00489514_m1	FAM
104	*Lamp2*	Mm00495267_m1	FAM
105	*Atg16l1*	Mm00513085_m1	FAM
106	*Atg5*	Mm01187303_m1	FAM
107	*Capn1*	Mm00482964_m1	FAM
108	*Tnf*	Mm00443258_m1	FAM
109	*Ctsb*	Mm01310506_m1	FAM
110	*Ern2*	Mm00469005_m1	FAM
111	*Eif2ak3*	Mm00438700_m1	FAM
112	*Atf6*	Mm01295319_m1	FAM
